# Association of endothelial and glycocalyx injury biomarkers with fluid administration, development of acute kidney injury, and 90-day mortality: data from the FINNAKI observational study

**DOI:** 10.1186/s13613-019-0575-y

**Published:** 2019-09-11

**Authors:** Nina Inkinen, Ville Pettilä, Päivi Lakkisto, Anne Kuitunen, Sakari Jukarainen, Stepani Bendel, Outi Inkinen, Tero Ala-Kokko, Suvi T. Vaara, the FINNAKI Study Group

**Affiliations:** 1 Department of Anesthesia and Intensive Care, Central Finland Central Hospital, Central Finland Health Care District, Keskussairaalantie 19 M rak 2krs, 40620 Jyväskylä, Finland; 2 Division of Intensive Care Medicine, Department of Anesthesiology, Intensive Care and Pain Medicine, University of Helsinki and Helsinki University Hospital, Helsinki, Finland; 3 Department of Clinical Chemistry, University of Helsinki and Helsinki University Hospital, Helsinki, Finland; 4 Minerva Foundation Institute for Medical Research, Helsinki, Finland; 5 Department of Intensive Care, University of Tampere and Tampere University Hospital, Tampere, Finland; 6 Department on Intensive Care Medicine, Kuopio University Hospital, Kuopio, Finland; 7 Department of Anaesthesia and Intensive Care Medicine, Turku University Hospital, Turku, Finland; 8 Research Group of Surgery, Anesthesiology and Intensive Care Medicine and Medical Research Center, Oulu University Hospital, Oulu University, Oulu, Finland; 9 Department of Intensive Care, Austin Hospital, Melbourne, Australia

**Keywords:** Biomarker, Glycocalyx, Sepsis, Fluid balance, Fluid resuscitation, Soluble thrombomodulin, Acute kidney injury

## Abstract

**Background:**

Injury to endothelium and glycocalyx predisposes to vascular leak, which may subsequently lead to increased fluid requirements and worse outcomes. In this post hoc study of the prospective multicenter observational Finnish Acute Kidney Injury (FINNAKI) cohort study conducted in 17 Finnish intensive care units, we studied the association of Syndecan-1 (SDC-1), Angiopoetin-2 (Ang-2), soluble thrombomodulin (sTM), vascular adhesion protein-1 (VAP-1) and interleukin-6 (IL-6) with fluid administration and balance among septic critical care patients and their association with development of acute kidney injury (AKI) and 90-day mortality.

**Results:**

SDC-1, Ang-2, sTM, VAP-1 and IL-6 levels were measured at ICU admission from 619 patients with sepsis. VAP-1 decreased (*p* < 0.001) and IL-6 increased (*p* < 0.001) with increasing amounts of administered fluid, but other biomarkers did not show differences according to fluid administration. In linear regression models adjusted for IL-6, only VAP-1 was significantly associated with fluid administration on day 1 (*p* < 0.001) and the cumulative fluid balance on day 5/ICU discharge (*p* = 0.001). Of 415 patients admitted without AKI, altogether 112 patients (27.0%) developed AKI > 12 h from ICU admission (AKI_>12 h_). They had higher sTM levels than patients without AKI, and after multivariable adjustment log, sTM level was associated with AKI_>12 h_ with OR (95% CI) of 12.71 (2.96–54.67), *p* = 0.001). Ninety-day non-survivors (*n* = 180; 29.1%) had higher SDC-1 and sTM levels compared to survivors. After adjustment for known confounders, log SDC-1 (OR [95% CI] 2.13 [1.31–3.49], *p* = 0.002), log sTM (OR [95% CI] 7.35 [2.29–23.57], *p* < 0.001), and log Ang-2 (OR [95% CI] 2.47 [1.44–4.14], *p* = 0.001) associated with an increased risk for 90-day mortality. Finally, patients who had high levels of all three markers, namely, SDC-1, Ang-2 and sTM, had an adjusted OR of 5.61 (95% CI 2.67–11.79; *p* < 0.001) for 90-day mortality.

**Conclusions:**

VAP-1 and IL-6 associated with fluid administration on the first ICU day. After adjusting for confounders, sTM was associated with development of AKI after 12 h from ICU admission. SDC-1, Ang-2 and sTM were independently associated with an increased risk for 90-day mortality.

## Background

Endothelial cells line the luminal side of all blood vessels. Glycocalyx is located on the top of the endothelium. It is a thin (few dozens of nanometers), complex layer with a framework consisting of glycoproteins and proteoglycans between the endothelial cell wall and flowing blood [1, 2]. It plays a key role in regulation of vascular permeability and blood cell–vessel interactions [1–3]. In critical illness, the body responds to stress by initiating sympathoadrenal activation, which leads to the release of inflammatory mediators, such as interleukin-6 (IL-6), and damage to the endothelium and glycocalyx, resulting in increased vascular permeability [4–6]. Several plasma biomarkers may be measured as indicators of glycocalyx [4, 7, 8] and endothelial injury [7–11].

We hypothesized that higher plasma levels of endothelial and glycocalyx biomarkers measured at intensive care unit (ICU) admission would indicate more severe endothelial and glycocalyx injury, more severe capillary leakage, and subsequently, higher amounts of administered fluid. Therefore, we primarily studied the association between glycocalyx (Syndecan-1; SDC-1) and endothelial injury biomarkers (Angiopoietin-2; Ang-2, soluble thrombomodulin; sTM, and vascular adhesion protein-1; VAP-1) and cytokine IL-6 (as marker of the severity of inflammation) and fluid administration among critically ill septic patients. Second, we aimed to study the association of these markers with acute kidney injury (AKI) and 90-day mortality.

## Methods

This was a post hoc laboratory study of the prospective multicenter observational Finnish Acute Kidney Injury (FINNAKI) cohort study conducted in 17 Finnish intensive care units in 2011–2012 [12]. The Ethics Committee of the Department of Surgery at the Helsinki University Hospital gave nationwide approval for the study (reference number: 18/13/03/02/2010) and accepted the use of deferred consent from the patient or next of kin with written informed consent obtained as soon as possible. The Finnish National Institute of Health and Welfare allowed data collection from medical records of deceased patients to avoid bias in the primary endpoint of the FINNAKI study (incidence and outcome of AKI). The current laboratory study includes only patients who or whose next of kin gave a written informed consent.

The FINNAKI study enrolled all adult emergency ICU admissions to study ICUs of any length and elective post-operative patients with an expected ICU stay longer than 24 h [12]. Exclusion criteria are presented in Additional file 1: Methods section of the 1.2, page 6. The current study included all FINNAKI study patients who were septic at ICU admission, had fluid balance data available for day 1, and a blood sample taken at ICU admission. FINNAKI study was observational and did not include fluid management protocol. Thus, all patients were treated according to the judgement of the treating clinician and the Surviving Sepsis Guidelines in place at that time [13].

### Data collection

We collected data on baseline chronic illnesses, ICU diagnoses, severity scores, and ICU treatment. Please see details in Additional file 1: 1.3, page 6.

### Definitions

We defined sepsis according to the American College of Chest Physicians/Society of Critical Care Medicine (ACCP/SCCM) definition [14]. Presence of AKI according to Kidney Disease: Improving Global Outcomes (KDIGO) criteria [15] considering creatinine (measured daily), hourly urine output, and use of renal replacement therapy (RRT) was screened until day 5 in the ICU. As a baseline creatinine, we used the most recent value from the previous year excluding the week preceding ICU admission according to the KDIGO guideline [15] and if it was not available, we estimated it using the modification of diet in renal disease (MDRD) equation assuming a GFR of 75 mL/min/1.73 m^2^ [16].

### Outcomes

Our primary outcome was association of SDC-1, Ang-2, sTM, VAP-1 and IL-6 with (a) fluid administration per hour within the first ICU day and (b) cumulative balance at ICU discharge or on day 5 whichever came first. Secondarily, we evaluated the independent associations of these biomarkers with development of AKI and 90-day mortality. From the AKI analysis, we excluded patients who were admitted with AKI (defined as AKI diagnosis made within 12 h of ICU admission) to study the temporal association of biomarkers and development of AKI [17].

### Calculation of fluid administration and balance

We prospectively collected data of the total amount of fluids administered to the patient daily including maintenance and resuscitation fluids, nutrition, blood products, drug infusions, and correspondingly, data about total fluid output (urine output, drainage fluids, bleeding, losses from gastrointestinal tract). A surrogate for evaporation (generally, 1000 mL daily for normothermic patients and additionally, an addition for each Celcius degree of fever per hour) was included in the fluid losses according to clinical practice. Fluid losses were subtracted from administered fluid to calculate fluid balance. The fluid data were collected from the ICU electronic patient records according to local ICUs fluid charting day. In most ICUs, the fluid charting day was a 24-h period starting from noon. Thus, the first study day was not a 24-h period for most of the patients (for example, if patient was admitted 8 p.m., the first fluid day was until next noon, altogether 16 h). Therefore, the duration of fluid administration and balance data collected for that day varied between 1 and 24 h. In the current analysis, we combined short fluid charting days (less than 2 h) to the administration and balance data of the subsequent day 1, and report here the administered fluid and balance normalized then to the total duration of fluid charting day that was between 2 and 26 h. Examples of these calculations are presented in Additional file 1: 1.4 page 7.

As an additional sensitivity analysis, fluid administration data from 44 patients from Helsinki University Hospital ICU were retrospectively extracted in 12-h blocks from the ICU data management system and were found to correlate well (rho 0.91, *p* < 0.001) with the day 0 administrations normalized to the duration of day 0 observation period.

Cumulative balance at ICU discharge or on day 5 (last data collection day) was normalized to patient weight at ICU admission and reported as a percentage.

### Laboratory samples and analyses

Plasma samples were collected at ICU admission (0 h) in lithium-heparin tubes, centrifuged, aliquoted, and frozen in − 80 °C until analyzed between April and October in 2017. To assess the temporal behavior of the markers, we analyzed sequential samples from 44 patients from Helsinki University Hospital ICU taken every 12 h until 36 h (40 patients had full series of samples from 0 to 36 h). Details of the laboratory analyses are presented in Additional file 1: 1.5 page 8.

### Statistical analyses

We report patients’ baseline characteristics using counts and percentages for categorical variables and medians with interquartile ranges (IQR) for continuous variables as data were non-normally distributed. To assess possible differences in patients grouped according to fluid input, AKI status, or survival, we compared continuous data using the Mann–Whitney *U* or Kruskal–Wallis tests and categorical data using the Fisher’s exact or Chi-squared tests, as appropriate.

We ran multivariable linear regression models examining the association between different biomarkers and fluid input or cumulative balance, controlling for the effect of interleukin-6. All biomarkers and fluid input on day 0 normalized to hours were log transformed. Three subjects were removed from the analyses due to having high influence (Cook’s Distance of over 4/(*n* − *p* − 1), where n is the number of subjects and *p* is the number of covariates). We compared sequential biomarker measurements from same patients with Friedman’s test.

We used multivariable logistic regression models, according to general practice concerning predictive models in critical care [18], to study whether biomarker levels were independently associated with the development of AKI after 12 h from admission (AKI_>12 h_) and 90-day mortality. We studied the effect of possible confounders (as listed in Table 1) in univariate models for development of AKI and 90-day mortality. We included all variables with a univariate *p* value less than 0.20 in multivariable models, however excluding variables that were clinically closely related (such as severity scores). Additionally, we adjusted for sex, as recommended [19]. Please see Additional file 1: 1.6 (page 8) about handling of missing data in regression models. Model fit was assessed with Hosmer–Lemeshow statistics.

**Table 1:** Patient characteristics

	Data available	All patients, *n* = 619	90d survivors, *n* = 439 (70.9%)	Non-survivors, *n* = 180 (29.1%)	*p* value
Age (years)	619	66 [55 to 75]	62 [52 to 73]	73 [62 to 80]	< 0.001
Sex; female	619	223 (36.0%)	154 (35.1%)	69 (38.3%)	0.462
Body mass index (kg/m^2^)	618	26.8 [23.6 to 30.1]	27.4 [24.1 to 30.9]	25.4 [23.0 to 27.9]	< 0.001
Comorbidities					
Hypertension	616	329 (53.2%)	229 (52.4%)	100 (55.9%)	0.477
Coronary artery disease or ASO	609	89 (14.4%)	55 (12.7%)	34 (19.3%)	0.043
Chronic heart failure	613	71 (11.5%)	38 (8.7%)	33 (18.5%)	0.001
COPD	612	79 (12.8%)	56 (12.9%)	23 (12.9%)	> 0.999
Chronic kidney disease (GFR < 60 mL/min/1.73 m^2^)	615	42 (6.8%)	23 (5.3%)	19 (10.7%)	0.021
Diabetes	619	158 (25.5%)	121 (27.6%)	37 (20.6%)	0.084
Baseline creatinine (µmol/L)	436	76 [61 to 92]	76 [61 to 91]	76 [62 to 98]	0.458
Pre-ICU chronic corticosteroid use	615	77 (12.4%)	41 (9.3%)	36 (20.0%)	< 0.001
Pre-ICU immunosuppression	614	56 (9.0%)	36 (8.2%)	20 (11.1%)	0.279
Pre-ICU radiocontrast	616	122 (19.7%)	93 (21.2%)	29 (16.1%)	0.150
Pre-ICU aminoglycoside	618	7 (1.1%)	2 (0.5%)	5 (2.8%)	0.013
Pre-ICU NSAID	582	94 (15.2%)	69 (15.7%)	25 (13.9%)	0.621
Pre-ICU amphotericin B	616	3 (0.5%)	0 [0.0%)	3 (1.7%)	0.024
Surgical admission	619	149 (24.1%)	115 (26.2%)	34 (18.9%)	0.062
Infection source					
CNS	619	18 (2.9%)	16 (3.6%)	2 (1.1%)	0.114
Lung	619	296 (47.8%)	209 (47.6%)	87 (48.3%)	0.929
GI tract	619	156 (25.2%)	108 (24.6%)	48 (26.7%)	0.611
Urinary tract	619	46 (7.4%)	34 (7.7%)	12 (6.7%)	0.737
Skin and soft tissue	619	55 (8.9%)	45 (10.3%)	10 (5.6%)	0.064
Other	619	19 (3.1%)	10 (2.3%)	9 (5.0%)	0.12
Time from hospital admission to ICU admission (h)	618	22 [9 to 59]	21 [8 to 53]	23 [11 to 74]	0.067
First lactate (mmol/L)^a^	536	1.80 [1.10 to 3.31]	1.60 [1.00 to 2.87]	2.20 [1.30 to 5.75]	< 0.001
First hematocrit (%)^a^	575	33 [29 to 37]	33 [29 to 37]	33 [28 to 37]	0.424
Vasopressor within first 24 h	619	438 (70.8%)	300 (68.3%)	138 (76.7%)	0.041
SOFA score at day 1	619	8 [6 to 10]	7 [6 to 9]	10 [7 to 13]	< 0.001
SAPS II score	619	42 [33 to 54]	39 [31 to 47]	54 [41 to 68]	< 0.001
SAPS II score without age and renal components	615	24 [18 to 32]	23 [17 to 29]	29 [22 to 29]	< 0.001
AKI (any severity)^b^	619	316 (51.1%)	203 (46.2%)	113 (62.8%)	< 0.001
AKI stage 1	619	122 (19.7%)	89 (20.3%)	33 (18.3%)	0.657
AKI stage 2	619	54 (8.7%)	40 (9.1%)	14 (7.8%)	0.641
AKI stage 3	619	140 (22.6%)	74 (16.9%)	66 (36.7%)	< 0.001
RRT for AKI during ICU stay	619	101 (16.3%)	52 (11.8%)	49 (27.2%)	< 0.001
Mechanical ventilation during ICU stay	619	404 (65.3%)	261 (59.5%)	143 (79.4%)	< 0.001
Fluid input on day 0 normalized to hours (mL)^c^	619	247 [173 to 363]	238 [167 to 337]	285 [184 to 417]	0.003
Cumulative balance %/weight^d^	579	1.06 [− 2.75 to 5.68]	0.15 [− 3.11 to 4.31]	4.13 [− 2.20 to 9.90]	< 0.001
ICU length of stay (days)	618	4 [2 to 7]	4 [2 to 6]	5 [2 to 9]	0.022

Categorical data reported as count (percentage) and continuous data as median [interquartile range, IQR]

*ASO* arteriosclerosis obliterans, *COPD* chronic obstructive pulmonal disease, *ICU* intensive care unit, *NSAID* non-steroidal anti-inflammatory drug, *CNS* central nervous system, *GI* gastrointestinal, *SOFA* sequential organ failure assessment considering all six organ systems, *SAPSII* Simplified Acute Physiology Score II, *AKI* acute kidney injury, *RRT* renal replacement therapy

^a^First value measured within first 6 h from ICU admission

^b^All AKI patients

^c^For details please see “Methods”

^d^Cumulative fluid accumulation at discharge/on day 5 during ICU stay as a percentage of body weight

To study whether a combination of biomarkers could identify a group of patients with especially high risk for AKI_>12 h_ and 90-day mortality [20], we built biomarker summary score that comprised biomarkers that were associated with the development of AKI and/or 90-day mortality in univariate models with *p* values less than 0.20 [21]. We categorized the biomarker levels into tertiles, and for each patient, calculated the total number of biomarkers (up to three) where the patient fell into the highest tertile. Next, we studied unadjusted and adjusted associations of the biomarker summary score with AKI_>12 h_ and 90-day mortality with logistic regression.

We set the significance level to 1% and considered *p* values < 0.01 as statistically significant. We conducted analyses with SPSS statistics 24 for Windows and SPSS 23 for Mac.

## Results

### Patients

We included 619 patients admitted to the ICU with sepsis or septic shock (Additional file 1: Figure S1). Altogether, 137 (22.1%) patients were admitted from operating room or postoperative intermediate care unit, 225 (36.3%) from emergency department, 197 (31.8%) from the wards and 60 (9.7%) from another ICU or intermediate care unit. The most common origins of sepsis were lungs in 296 (47.8%) and gastrointestinal tract in 156 (25.2%) patients (Table 1). Biomarker levels at ICU admission according to the presence of baseline chronic illnesses are presented in Additional file 1: 2.1, page 10 and Table S1.

### Fluid administration, balance and biomarker levels

**Fig. 1:**
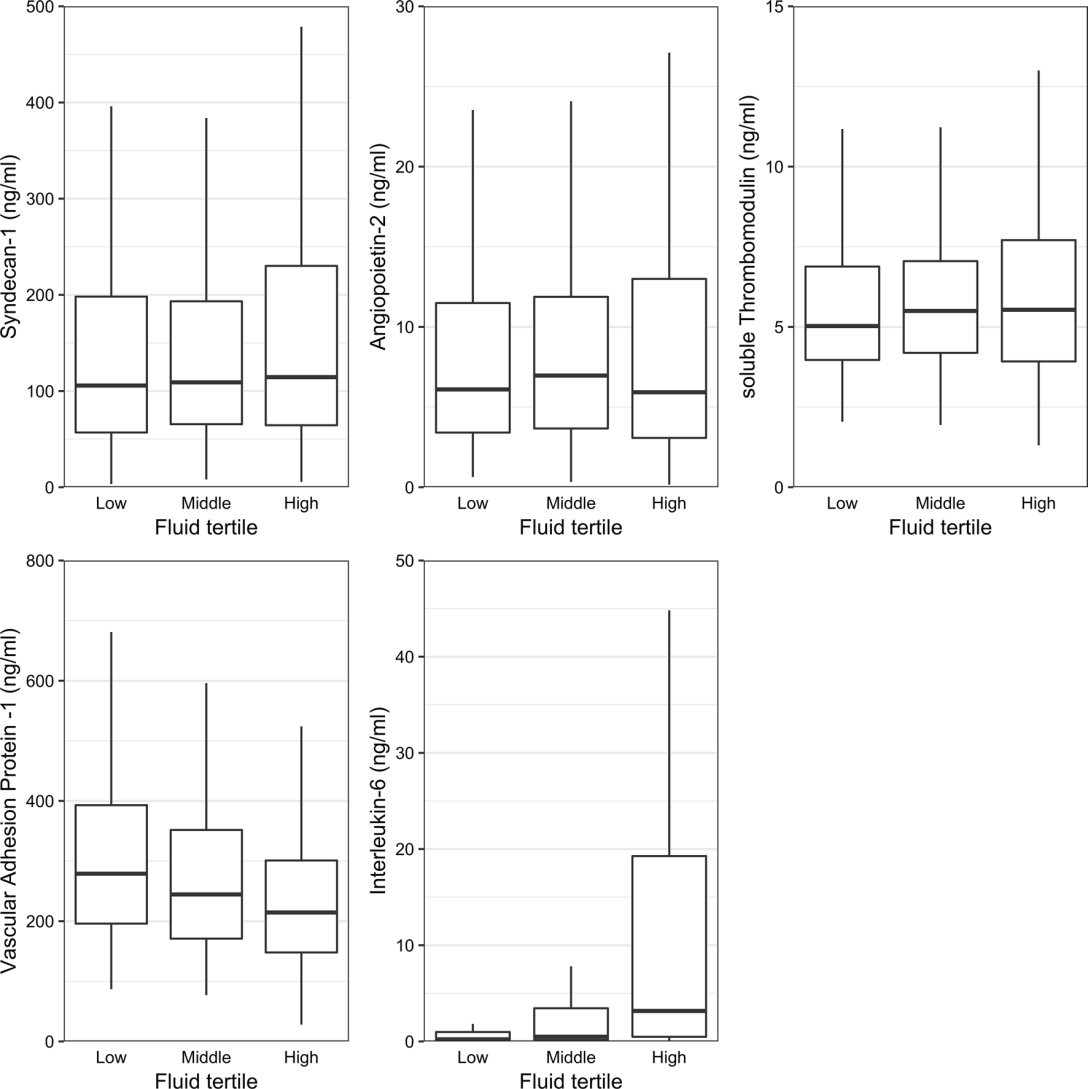
Biomarker levels at ICU admission according to fluid administration tertiles. Fluid tertile 1: < 201 mL/h. Fluid tertile 2: 202–314 mL/h. Fluid tertile 3: 315–2824 mL/h. Between fluid tertiles 1 and 2 IL-6 *p* value < 0.001, all other biomarkers’ *p* values > 0.010; between fluid tertiles 1 and 3 VAP-1 *p* value < 0.001 and IL-6 *p* value < 0.001, all other biomarkers’ *p* values > 0.010; between fluid tertiles 2 and 3 VAP-1 *p* value = 0.003 and IL-6 *p* value < 0.001, all other biomarkers’ *p* values > 0.010

During the first ICU day, the patients received a median of 247 mL/h (IQR [173–363] mL/h) fluids. Divided into tertiles according to fluid administration, less than 201 mL/h fluid was administered in the 1st tertile, 202–314 mL/h in the 2nd tertile, and 315–2824 mL/h in the 3rd tertile. In all 619 patients VAP-1 levels were significantly lower in the lowest fluid administration tertile (*p* < 0.001) and in the middle tertile (*p* = 0.003) compared to the highest tertile, and IL-6 levels higher (*p* < 0.001) in the highest fluid administration tertile compared to the middle or lowest tertile (Fig. 1, Table 2). SDC-1, Ang-2, or sTM levels did not differ between the fluid administration tertiles (Fig. 1, Table 2). Correlations between biomarkers are presented in Additional file 1: Table S2. All other biomarkers showed significant correlation with each other except (1) VAP-1 between SDC-1, (2) Ang-2 and sTM between IL-6. The strongest correlation was between SDC-1 and sTM with rho of 0.406 (*p* < 0.001). IL-6 and VAP-1 correlated negatively with each other (Additional file 1: Table S2). In linear regression models adjusted for IL-6, only VAP-1 was significantly associated with fluid administration on day 1 (ß [95% CI] − 0.139 [− 0.213 to − 0.064], *p* < 0.001) and the cumulative fluid balance on day 5/ICU discharge (ß [95% CI] − 0.130 [− 0.209 to − 0.052], *p* = 0.001) (Additional file 1: Tables S3 and S4). In the sequential samples subanalysis including 40 patients with biomarkers measured every 12 h until 36 h available, SDC-1 values were increasing over time (*p* < 0.001), IL-6 decreased (*p* < 0.001), and others did not show significant differences (Additional file 1: Figure S2).

**Table 2:** Biomarker levels and fluid administration

Biomarker level (ng/mL) at 0 h (median [IQR])	Fluid administration			
	1st tertile, *n* = 205 (4–201 mL/h)	2nd tertile, *n* = 206 (202–314 mL/h)	3rd tertile, *n* = 208 (315–2824 mL/h)	*p* value
Syndecan-1	105.70 [56.65–205.20]	109.05 [64.93–194.25]	114.45 [64.08–231.85]	0.276
Angiopoietin-2	6.10 [3.40–11.64]	6.97 [3.63–11.98]	5.92 [3.06–13.31]	0.669
Soluble thrombomodulin	5.03 [3.94–6.93]	5.50 [4.18–7.07]	5.53 [3.91–7.72]	0.282
Vascular adhesion protein-1	279.00 [195.50–395.50]	244.50 [170.75–355.25]	214.50 [148.00–305.00]	< 0.001
Interleukin-6	0.22 [0.07–1.00]	0.49 [0.16–0.36]	3.17 [0.45–19.51]	< 0.001

*IQR* interquartile range

### Biomarkers and organ failures and severity of sepsis

Patients whose SOFA scores decreased from first ICU day to ICU day 3 (*n* = 491) had significantly lower SDC-1 (*p* = 0.003) and IL-6 (*p* = 0.001) levels at ICU admission compared to those with worsening or stable SOFA scores (*n* = 127). Other biomarkers did not correlate with changes in SOFA scores.

### Biomarkers and AKI

Altogether, 316 (51.1%) patients had AKI. Of them, 189 (59.8%) were diagnosed based on creatinine criterion only, 69 (21.8%) based on urine output criterion only, 28 (8.9%) had both creatinine and urine output criteria positive, and in 30 (9.5%) patients commenced RRT before fulfilling other criteria. Of AKI patients, 204 (64.6%) were admitted with AKI (AKI diagnosed 0–12 h from ICU admission), whereas 112 (35.4%) were diagnosed more than 12 h from ICU admission. Of these ,112 AKI_>12 h_ patients 59 (52.7%) had stage 1, 16 (14.3%) had stage 2, and 37 (33.0%) had stage 3 AKI according to the KDIGO criteria. All other biomarkers except VAP-1 were significantly higher in all AKI patients compared to non-AKI patients (Additional file 1: Table S5). Among AKI_>12 h_ patients SDC-1 and sTM were significantly higher compared to non-AKI patients (Table 3). After adjusting for possible confounders and IL-6 levels, sTM was significantly associated with AKI_>12 h_ (Table 4). Patients with the maximum biomarker summary score of 3 had an OR of 4.24 (95% CI 1.64–10.96, *p* = 0.003) for the development of AKI as compared to patients with the minimum biomarker summary score of 0 (Table 4).

**Table 3:** Biomarker levels in all patients and according to development of acute kidney injury

Biomarker level (ng/mL) at 0 h (median [IQR])	All patients (*n* = 619)	No AKI (*n* = 303)	AKI > 12 h (*n* = 112)	*p* value
Syndecan-1	109.00 [62.30–215.40]	91.40 [55.30–157.30]	125.25 [67.60–232.68]	0.004
Angiopoietin-2	6.31 [3.39–12.13]	5.53 [2.99–9.92]	7.44 [4.29–13.22]	0.028
Soluble Thrombomodulin	5.30 [4.02–7.13]	4.61 [3.69–5.97]	5.84 [4.37–7.94]	< 0.001
Vascular adhesion protein-1	241.00 [171.00–340.00]	255.00 [177.00–356.00]	241.50 [170.75–340.00]	0.889
Interleukin-6	0.57 [0.14–4.74]	0.36 [0.12–3.06]	0.83 [0.17–5.10]	0.011

AKI > 12 h; AKI diagnosed over 12 h from ICU admission to day 5 or ICU discharge, if earlier

**Table 4:** Unadjusted and adjusted single and multivariable biomarker logistic regression models for the development of acute kidney injury

Single biomarker models						
Biomarker	Unadjusted OR (95% CI)	*p* value	IL-6 adjusted^a^ OR (95% CI)	*p* value	Multivariable adjusted^b^ OR (95% CI)	*p* value
Log Angiopoietin-2	2.66 (1.53–4.63)^c^	0.001	2.40 (1.37–4.21)	0.002	1.96 (1.05–3.66)	0.034
Log soluble thrombomodulin	30.72 (8.37–112.67)	< 0.001	29.55 (7.87–110.89)	< 0.001	12.71 (2.96–54.67)	0.001
Log Syndecan-1	1.98 (1.15–3.42)	0.014	1.90 (1.10–3.30)	0.022	1.49 (0.80–2.77)	0.212
Log vascular adhesion protein 1	1.07 (0.40–2.88)	0.891	1.66 (0.59–4.71)	0.340	0.93 (0.28–3.07)	0.907

Biomarker summary score^c^						
Score-patients with AKI no/total to (%)	Unadjusted OR (95% CI)	*p* value	IL-6 adjusted^a^ OR (95% CI)	*p* value	Multivariable adjusted^b^ OR (95% CI)	*p* value
0–36/195 (18.5%)	Reference	–	Reference	–	Reference	–
1–32/121 (26.4%)	1.59 (0.92–2.73)	0.095	1.47 (0.85–2.54)	0.169	1.50 (0.83–2.70)	0.176
2–28/72 (38.9%)	2.81 (1.55–5.10)	0.001	2.73 (1.49–4.97)	0.001	2.11 (1.09–4.11)	0.027
3–16/27 (59.3%)	6.42 (2.75–15.01)	< 0.001	5.96 (2.53–14.03)	< 0.001	4.24 (1.64–10.96)	0.003

Hosmer–Lemeshow test non-significant in all analyses

*AKI* acute kidney injury, *IL-6* interleukin-6

^a^Adjusted for log-transformed interleukin-6 value at ICU admission

^b^Adjusted for age, sex, body mass index, pre-existing chronic kidney disease, pre-existing diabetes mellitus, presence of urinary tract infection, presence of skin or soft tissue infection, Simplified Acute Physiology Score (SAPS) II without points for age and renal components, need for vasoactive medication within first 24 h, first lactate in ICU, log-transformed interleukin-6 value at ICU admission. Total number of patients in the model was 415. ^b^Including Angiopoietin-2, soluble thrombomodulin and Syndecan-1

^c^An example of the interpretation of the OR of log-transformed biomarkers: the OR for development for AKI for Log Angiopoietin-2 is 2.66, so when untransformed Angiopoietin-2 levels grow by a factor of 10, the odds for AKI increase by a factor of 2.66

### 90-day mortality

By day 90, 180 (29.1%) patients were deceased. Non-survivors had higher SDC-1 and sTM levels than survivors (Table 5). After adjusting for multiple confounders, SDC-1, Ang2, and sTM were independently associated with an increased risk for 90-day mortality (Table 6). Altogether 65 patients had the maximum score of 3 in the biomarker summary score, and 35 (53.8%) of them were deceased by day 90. As compared to patients with the minimum biomarker summary score of 0, patients with the maximum score had the adjusted odds of OR 5.61 (95% CI 2.67–11.79, *p* < 0.001) for 90-day mortality (Table 6).

**Table 5:** Biomarker levels in all patients and according to 90-day mortality

Biomarker level (ng/mL) at 0 h (median [IQR])	All patients (*n* = 619)	90-day survivors (*n* = 439)	90-day non-survivors (*n* = 180)	*p* value
Syndecan-1	109.00 [62.30–215.40]	98.10 [58.40–180.00]	157.05 [72.50–310.80]	< 0.001
Angiopoietin-2	6.31 [3.39–12.13]	5.92 [3.30–11.26]	7.60 [4.01–17.44]	0.013
Soluble thrombomodulin	5.30 [4.02–7.13]	5.08 [3.88–6.81]	5.90 [4.37–8.19]	< 0.001
Vascular adhesion protein-1	241.00 [171.00–340.00]	239.00 [171.00–339.00]	253.00 [168.25–340.00]	0.676
Interleukin-6	0.57 [0.14–4.74]	0.54 [0.13–4.25]	0.67 [0.15–6.51]	0.147

**Table 6:** Unadjusted and adjusted single and multivariable biomarker models for 90-day mortality

Single biomarker models						
Biomarker	Unadjusted OR (95% CI)	*p* value	IL-6 adjusted^a^ OR (95% CI)	*p* value	Multivariable adjusted^b^ OR (95% CI)	*p* value
Log Angiopoietin-2	1.69 (1.12–2.55)	0.012	1.57 (1.03–2.38)	0.035	2.47 (1.44–4.14)	0.001
Log soluble thrombomodulin	2.36 (1.58–3.53)	< 0.001	2.27 (1.52–3.40)	< 0.001	7.35 (2.29–23.57)	0.001
Log Syndecan-1	6.45 (2.58–16.09)	< 0.001	5.94 (2.36–14.91)	< 0.001	2.13 (1.31–3.49)	0.002
Log vascular adhesion protein 1	0.99 (0.47–2.09)	0.980	1.26 (0.58–2.75)	0.565	2.02 (0.73–5.57)	0.177

Biomarker summary score^a^						
Score-90-day non-survivors no/total to (%)	Unadjusted OR (95% CI)	*p* value	IL-6 adjusted^a^ OR (95% CI)	*p* value	Multivariable adjusted^b^ OR (95% CI)	*p* value
0–57/249 (22.9%)	Reference	–	Reference	–	Reference	–
1–49/187 (26.2%)	1.20 (0.77–1.86)	0.425	1.17 (0.75–1.81)	0.500	1.19 (0.70–2.01)	0.530
2–39/118 (33.1%)	1.66 (1.03–2.70)	0.040	1.60 (0.98–2.61)	0.058	1.93 (1.07–3.47)	0.029
3–35/65 (53.8%)	3.93 (2.22–6.95)	< 0.001	3.69 (2.07–6.57)	< 0.001	5.61 (2.67–11.79)	< 0.001

Hosmer–Lemeshow test non-significant in all analyses

*IL-6* interleukin-6

^a^Adjusted for log-transformed interleukin-6 value at ICU admission

^b^Adjusted for age, sex, body mass index, pre-existing arteriosclerosis, chronic heart failure, chronic kidney disease, diabetes mellitus, chronic corticosteroid use, operative admission, skin or soft tissue infection, central nervous system infection, other infection source, need for vasoactive medication within first 24 h, Simplified Acute Physiology Score (SAPS) II without points for age and renal components, first lactate in ICU, acute kidney injury within first 5 days in the ICU, fluid accumulation percentage of baseline weight within first 5 days in ICU, mechanical ventilation during ICU stay, and log-transformed interleukin-6 value at ICU admission. Total number of patients in the model was 619

^c^Including Angiopoietin-2, soluble thrombomodulin and Syndecan-1, each value in the highest tertile for each biomarker yielding a subscore of 1

## Discussion

We found VAP-1 and IL-6 to be associated with the amount of administered fluid on the first ICU treatment day and other fluid balance-related variables; however, the other investigated markers did not differ according to fluid administration. Only sTM remained associated with the development of AKI after adjusting for known confounders. Finally, Ang-2, SDC-1, and sTM were associated with an increased risk for 90-day mortality, also after adjustments. Our results demonstrate that injury to glycocalyx and endothelium present at ICU admission is associated with an increased risk for AKI and mortality.

We observed roughly comparable plasma biomarker levels to previous studies [8, 22, 23]. Previously, Ang-2 has been shown to correlate with the first 6-h balance, but not with the 24-h balance [24]. Our results corroborate this finding. However, we did not find significant differences in SDC-1 levels in terms of fluid administration or balance, in contrast with a previous study that found SDC-1 to be associated with fluid balance over the first 24 h [25]. That study measured SDC-1 levels on the second ICU day, whereas we obtained samples right after ICU admission. A novel finding in our study was the association of higher IL-6 and lower VAP-1 levels with higher amount of received fluids. As more severely ill patients have higher IL-6 levels [26], and typically receive also more fluids [27], the finding regarding IL-6 was to be expected. The finding regarding VAP-1 is surprising, as one would have expected higher VAP-1 levels with higher fluid administration and severity of illness. However, the transmembrane form of VAP-1 facilitates leukocyte rolling and transmigration, whereas the soluble form acts as a primary serum amine oxidase [28]. Possibly, in the most severely ill septic patients, the shedding of transmembrane form of VAP-1 into soluble form is reduced due to greater needs of the transmembrane form. Moreover, the plasma levels do not necessarily reflect the tissue levels of a biomarker. Finally, dilution of VAP-1 with increasing fluid balance cannot be excluded.

sTM was independently associated with the development of AKI, as in a recent retrospective observational study among 514 septic patients [29] and a multicenter prospective observational study among 80 ICU patients [30]. Moreover, we demonstrated that an increase in sTM levels occurred already before AKI onset, unlike in the largest previous study [29]. Interestingly, we found Ang-2 to be significantly associated with AKI in the univariate analysis but not in the adjusted model. A previous study reported an unadjusted association of AKI defined by the KDIGO creatinine criterion and Ang-2 levels [24]. Higher SDC-1 values have been previously observed to be associated with AKI in a study among 175 septic patients [31]. Albeit the SDC-1 levels in this study were higher among AKI_>12 h_ patients, SDC-1 was not independently associated with AKI.

Our study corroborates previous results on the associations between higher SDC-1, Ang-2 and sTM plasma levels and the severity of critical illness and mortality [9, 22, 25, 31–33]. A multicenter trial in acute respiratory distress syndrome (ARDS) patients reported a positive correlation between sTM plasma levels and mortality, but found no difference in sTM levels between conservative and liberal fluid management groups [34]. Moreover, a recent study comparing early goal-directed therapy, standard therapy, and protocolized therapy found no difference in endothelial injury biomarkers including sTM and Ang-2 between the treatment arms, but higher Ang-2 and STM plasma levels were associated with 60-day mortality [32]. We found an independent association between Ang-2, sTM, and SDC-1 and 90-day mortality. Moreover, patients who had high levels of all these three markers had a fivefold adjusted OR for 90-day mortality compared to those in whom all these markers were low.

Given the multiple roles of the glycocalyx and endothelium in regulating vascular permeability and leukocyte adhesion [1–4], our results on increased mortality are biologically plausible. A recent animal study in experimental sepsis found fluid resuscitation (compared to no resuscitation) to increase atrial natriuretic peptide (ANP) levels and lead to glycocalyx injury [35]. Furthermore, fluid resuscitation group exhibited higher vasopressor requirements, potentially due to glycocalyx damage [35]. Additionally, hypervolemia has been found to increase glycocalyx shedding in an experimental volume loading study in surgical patients [36]. Taken together, these data suggest that some of the typical interventions used in sepsis resuscitation may aggravate glycocalyx and endothelial injury. Our results add to the increasing body of evidence highlighting the importance of endothelial and glycocalyx injury in the pathway of adverse outcomes and call for studies assessing novel means in avoiding and alleviating injury to endothelium and glycocalyx.

Our study has some strengths and limitations. This was a post hoc study from a large prospective multicenter study with good generalizability to other critically ill septic patient cohorts. Moreover, we used the full set of KDIGO AKI criteria, and collected detailed and comprehensive data about clinical variables and risk factors for AKI. Most of the previous studies have used only the creatinine criterion for AKI [22–24, 31]. As a limitation, first, our study collected data about fluid administration and balance in 24-h blocks, which may not be granular enough to distinguish the fluid resuscitation phase. However, we extracted the fluid administration data of 44 patients in 12-h blocks from the ICU data management system as a sensitivity analysis, and found it to correlate well with the ICU admission day fluid administration normalized to the duration of observation period. Second, we did not have data regarding the pre-ICU fluid therapy, which may have affected plasma biomarker levels at ICU admission and may explain why we could not show a connection between other biomarkers than VAP-1 and IL-6 and fluid load. However, some studies have suffered from the same limitation [25]. Third, we had to exclude 36 deceased patients from the analysis because of absent consent. Nevertheless, percentage of these patients of the cohort was low (5.5%), and thus, unlikely to cause significant bias. Fourth, this study was observational and had no protocol concerning hemodynamic monitoring or fluid responsiveness. Therefore, hemodynamic monitoring was performed and fluids were administered according to the treating clinician and standards of each participating ICU following the Surviving Sepsis Guidelines [13] in place that time. Fifth, we defined sepsis according to the American College of Chest Physicians/Society of Critical Care Medicine (ACCP/SCCM) definition [14]. Due to the structure of data collection, classifying patients retrospectively according to the sepsis-3 definition was not possible. Sixth, fluid balance was calculated with inputs and outputs since weighting of all study participants was not possible. However, it is not obvious that regular body weight measures would be more precise to estimate fluid balance than fluid balance calculations [37]. Seventh, we used data-driven approach supplemented with clinical discretion in selecting confounders for the logistic regression analyses, albeit a propensity score analysis might have been able to mitigate confounding better [38]. Finally, some unknown confounders might have been missing.

## Conclusions

We found VAP-1 and IL-6, but not the other markers, to be associated with fluid administration on the first ICU day. Supporting previous results, sTM was independently associated with the development of AKI. Finally, Ang-2, sTM, and SDC-1 were independently associated with an increased risk for 90-day mortality. These findings support the importance of endothelial injury in the pathophysiology of sepsis-associated AKI and highlight the role of the glycocalyx and endothelium in the pathway of adverse outcomes.

## Supplementary information

**Additional file 1: Figure S1.** Flow chart. **Table S1**. Biomarker levels and chronic diseases. **Figure S2**. Sequential samples subanalysis including 40 patients with biomarkers measured every 12hrs until 36 hrs. **Table S2**. Correlation between biomarkers. **Table S3**. Multivariable linear regression models predicting log Fluid input on Day 0 normalized to hours. **Table S4**. Multivariable linear regression models predicting Cumulative balance %/weight. **TableS5**. Biomarker levels and acute kidney injury.

## Data Availability

The datasets analyzed during this study are available from the corresponding author for a reasonable request.

## Abbreviations

AKI: acute kidney injury
AKI_>12 h_: development of AKI after 12 h from admission
Ang-2: angiopoietin-2
ANP: atrial natriuretic peptide
ICU: intensive care unit
IL-6: interleukin-6
KDIGO: Kidney Disease Improving Global Outcomes
SAPS II: Simplified Acute Physiology Score II
SDC-1: syndecan-1
SOFA: sequential organ failure assessment
sTM: soluble thrombomodulin
VAP-1: vascular adhesion protein-1
