# Supplementary material for: Association of endothelial and glycocalyx injury biomarkers with fluid administration, development of acute kidney injury, and 90-day mortality: data from the FINNAKI observational study
Source: Ann Intensive Care. 2019 Sep 11;9:103. doi: 10.1186/s13613-019-0575-y (PMC6738365; doi:10.1186/s13613-019-0575-y)
Supplement: Supplementary file 1 — Additional file 1: Figure S1. Flow chart. Table S1. Biomarker levels and chronic diseases. Figure S2. Sequential samples subanalysis including 40 patients with biomarkers measured every 12hrs until 36 hrs. Table S2. Correlation between biomarkers. Table S3. Multivariable linear regression models predicting log Fluid input on Day 0 normalized to hours. Table S4. Multivariable linear regression models predicting Cumulative balance %/weight. TableS5. Biomarker levels and acute kidney injury. [file 13613_2019_575_MOESM1_ESM.docx]

**ADDITIONAL FILE**

Association of endothelial biomarkers with fluid administration, development of acute kidney injury, and 90-day mortality – data from the FINNAKI observational cohort study

*Nina Inkinen, Ville Pettilä, Päivi Lakkisto, Anne Kuitunen, Sakari Jukarainen, Stepani Bendel, Outi Inkinen, Tero Ala-Kokko, Suvi Vaara*

The FINNAKI Study group

The following are the members of the **FINNAKI study group**: **Central Finland Central Hospital**: Raili Laru-Sompa, Anni Pulkkinen, Minna Saarelainen, Mikko Reilama, Sinikka Tolmunen, Ulla Rantalainen, Marja Miettinen **East Savo Central Hospital**: Markku Suvela, Katrine Pesola, Pekka Saastamoinen, Sirpa Kauppinen **Helsinki University Central Hospital**: Ville Pettilä, Kirsi-Maija Kaukonen, Anna-Maija Korhonen, Sara Nisula, Suvi Vaara, Raili Suojaranta-Ylinen, Leena Mildh, Mikko Haapio, Laura Nurminen, Sari Sutinen, Leena Pettilä, Helinä Laitinen, Heidi Syrjä, Kirsi Henttonen, Elina Lappi, Hillevi Boman **Jorvi Central Hospital**: Tero Varpula, Päivi Porkka, Mirka Sivula, Mira Rahkonen, Anne Tsurkka, Taina Nieminen, Niina Pirttinen **Kanta-Häme Central hospital**: Ari Alaspää, Ville Salanto, Hanna Juntunen, Teija Sanisalo **Kuopio University Hospital**: Ilkka Parviainen, Ari Uusaro, Esko Ruokonen, Stepani Bendel, Niina Rissanen, Maarit Lång, Sari Rahikainen, Saija Rissanen, Merja Ahonen, Elina Halonen, Eija Vaskelainen **Lapland Central Hospital**: Meri Poukkanen, Esa Lintula, Sirpa Suominen **Länsi-Pohja Central Hospital**: Jorma Heikkinen, Timo Lavander, Kirsi Heinonen, Anne-Mari Juopperi **Middle Ostrobothnia Central Hospital**: Tadeusz Kaminski, Fiia Gäddnäs, Tuija Kuusela, Jane Roiko **North Karelia Central Hospital**: Sari Karlsson, Matti Reinikainen, Tero Surakka, Helena Jyrkönen, Tanja Eiserbeck, Jaana Kallinen **Oulu University Hospital**: Tero Ala-Kokko, Jouko Laurila, Sinikka Sälkiö **Satakunta Hospital District**: Vesa Lund, Päivi Tuominen, Pauliina Perkola, Riikka Tuominen, Marika Hietaranta, Satu Johansson **South Karelia Central Hospital**: Seppo Hovilehto, Anne Kirsi, Pekka Tiainen, Tuija Myllärinen, Pirjo Leino, Anne Toropainen **Tampere University Hospital**: Anne Kuitunen, Jyrki Tenhunen, Ilona Leppänen, Markus Levoranta, Sanna Hoppu, Jukka Sauranen, Atte Kukkurainen, Samuli Kortelainen, Simo Varila **Turku University Hospital**: Outi Inkinen, Niina Koivuviita, Jutta Kotamäki, Anu Laine

**Corresponding author:**

Nina Inkinen

Address: Department of Anesthesia and Intensive Care, Central Finland Central Hospital, Central Finland Health Care District, Keskussairaalantie 19 M rak 2krs, 40620 Jyväskylä, Finland. Phone: +358 14 269 5375, e-mail: nina.inkinen@ksshp.fi

**Table of Contents**

1. Supplementary methods

1.1 Biomarker Selection

1.2 FINNAKI study exclusion criteria

1.3 FINNAKI study data collection

1.4 Fluid administration data calculation examples

1.5 Laboratory analyses

1.6 Handling of missing data in regression models

1. Supplementary results

2.1 Biomarker levels and chronic diseases

2.2 Additonal file Figure S1. Flow chart

2.3 Additional file Table S1. Biomarker levels and chronic diseases

2.4 Additional file Figure S2. Sequential samples subanalysis including 40 patients with biomarkers measured every 12 hrs until 36 hrs.

2.5 Additional file Table S2. Correlation between biomarkers

2.6 Additional file Table S3. Multivariable linear regression models predicting log Fluid input on Day 0 normalized to hours

2.7 Additional file Table S4. Multivariable linear regression models predicting Cumulative balance %/weight

2.8 Additional file Table S5. Biomarker levels and acute kidney injury

Supplemental methods

### 1.1 Biomarker Selection

#### Syndecan-1 (SDC-1)

SDC-1 is a heparin sulphate proteoglycan widely expressed on endothelial surfaces (1). It is involved with several mediators of disease pathogenesis. It can, for example, modulate leukocyte recruitment and microbial attachment and entry (1). Higher SDC-1 levels have been noticed to correlate with worse outcomes in trauma patients (2) or organ failure (3), and need for intubation in septic patients (4).

#### Angiopoietin-2 (Ang-2)

Ang-2 is a protein secreted from the endothelium by inflammatory stimulus. It mediates breakdown of the endothelial glycocalyx and increases vascular permeability (5). Ang-2 is an antagonist to the vascular-associated receptor tyrosine kinase Tie2 which controls the promotion of microvascular barrier function and anti-inflammatory properties (5). High levels of Ang-2 have been measured in septic patients with impaired oxygenation and furthermore, it has been shown to provoke vascular hyperpermeability and pulmonary congestion in mice (6) and to associate with increased mortality in critically ill patients (7,8). Ang-2 has also been reported to correlate positive with fluid balance in septic patients (9,10).

#### Soluble thrombomodulin (sTM)

sTM is the circulating form of glycoprotein thrombomodulin (TM) which acts as thrombin receptor on the surface of endothelial cell. TM is cleaved from the endothelial cell to become sTM when the endothelium is injured (11). Elevated sTM level has been shown to predict AKI (9) and 90-day mortality (3) in septic patients. Higher sTM level has also been reported to correlate with fluid balance in ARDS patients (12).

#### Vascular adhesion protein -1 (VAP-1)

VAP-1, a transmembrane glycoprotein, is an adhesion molecule acting in leukocyte rolling, adhesion and transmigration (13). It is manifested on the vascular endothelial cells, smooth muscle and adipocytes (14). VAP-1 acts as an ectoenzyme, a cell surface molecule, and it belongs to primary amino oxidases. It catalyzes primary amines oxidative deamination to aldehydes and that reaction produces also hydrogenperoxide and ammonium. Inhibition of VAP-1 have been noticed to deteriorate inflammatory reactions in animal models by reducing the amount of infiltrating leucocytes (14).

### 1.2 FINNAKI study exclusion criteria

The FINNAKI-study exclusion criteria were 1) age under 18, 2) chronic dialysis, 3) received renal replacement therapy (RRT) under previous ICU admission included in the FINNAKI study, 4) organ donor, 5) admitted for intermediate care, 6) transfer from another study ICU with completed study observation period, and 7) no permanent residency in Finland or insufficient language skills for informed consent.

### 1.3 FINNAKI study data collection

We collected information of patient characteristics, chronic illnesses, fluid administration and balance, source of infection, physiological and laboratory data, diagnoses, severity scores (calculated in 24-hour periods from ICU admission) and given ICU treatment in case report forms. Data were recorded from admission to day 5 if patient was still located in the ICU. These data were supplemented by data from Finnish Intensive Care Consortium database that included for example data of illness severity scores and ICU diagnoses. Statistics Finland provided the information of patient survival at 90 days from admission.

### 1.4 Fluid administration data calculation examples

If day 0 (admission day) was shorter than 2 hours, data about administered fluids and balance were combined to day 1 data.

Example 1.

At ICU, the fluid day begins at 00:00 am. Patient is admitted to the ICU at day 0 at 11:15 pm. Fluid data were collected from hour 11pm (the beginning of the calculation period was rounded to the nearest even hour) to 12pm and added to day 1 fluid data (1 hour data+ 24 hours data =25 hours data) and then divided to 25 and then multiplied to 24.

Example 2.

The fluid day begins at 00:00 am. Patient is admitted to the ICU at 03:40 pm. Day 0 fluid data were collected from hour 04:00 pm (because of calculation period rounding to the nearest even hour), divided to 8 and then multiplied to 24.

### 1.5 Laboratory analyses

Biomarker levels were measured using following commercial Enzyme-Linked Immunosorbent Assay (ELISA) assays: SDC-1 (Human CD138 ELISA kit, Diaclone, Besancon, France), sTM (Human Thrombomodulin Quantikine ELISA, R&D systems, UK), VAP-1 (Human VAP-1 Quantikine ELISA, R&D systems), Ang-2 (Human Angiopoietin-2 Quantikine ELISA, R&D systems) and IL-6 (Human IL-6 Quantikine ELISA, R&D systems). Assays were performed according to manufacturers’ instructions. To exclude possible interfering factors, high IL-6 results were confirmed using another ELISA assay that has different antibodies against IL-6 (IL-6 human ELISA kit, Cayman Chemical, Ann Arbor, MI, USA).

### 1.6 Handling of missing data in regression models

For the logistic regression models, missing lactate values (N=83; 13.4%), cumulative fluid accumulation at discharge/ on day 5 during ICU stay as a percentage of body weight (N=40; 6.5%), and SAPS II scores (N=4; 0.6%) were imputed with the median value and one missing weight value was imputed with the median of same sex. Individuals with missing values of pre-existing arteriosclerosis (N=10), heart failure (N=6), and chronic kidney disease (N=4) were imputed as not having the diseases.

## Supplemental Results

Additional file Figure S1. Flow chart

Additional file Table S1: Biomarker levels and chronic diseases

Additional Figure S2. Sequential samples subanalysis including 40 patients with biomarkers measured every 12 hrs until 36 hrs.

Additional file Table S2. Correlation between biomarkers

Additional file Table S3. Multivariable linear regression models predicting log Fluid input on Day 0 normalized to hours

Additional file Table S4. Multivariable linear regression models predicting Cumulative balance %/weight

Additional file Table S5. Biomarker levels and acute kidney injury

2.1 Biomarker levels and chronic diseases

All markers except VAP-1 correlated with the day 1 SOFA score. SDC-1 levels were higher in patients with chronic coronary and/or peripheral artery disease and IL-6 levels lower in COPD patients. VAP-1 levels were significantly higher in chronic heart failure patients, CKD patients, and diabetics whose sTM levels were also significantly higher compared to non-diabetics (Additional file Table S1). Patients on chronic corticosteroid medication or other immunosuppressive medication before ICU admission had significantly lower VAP-1 levels compared to other patients (Additional file Table S1.)

2.2 Additional file Figure S1. Flow chart


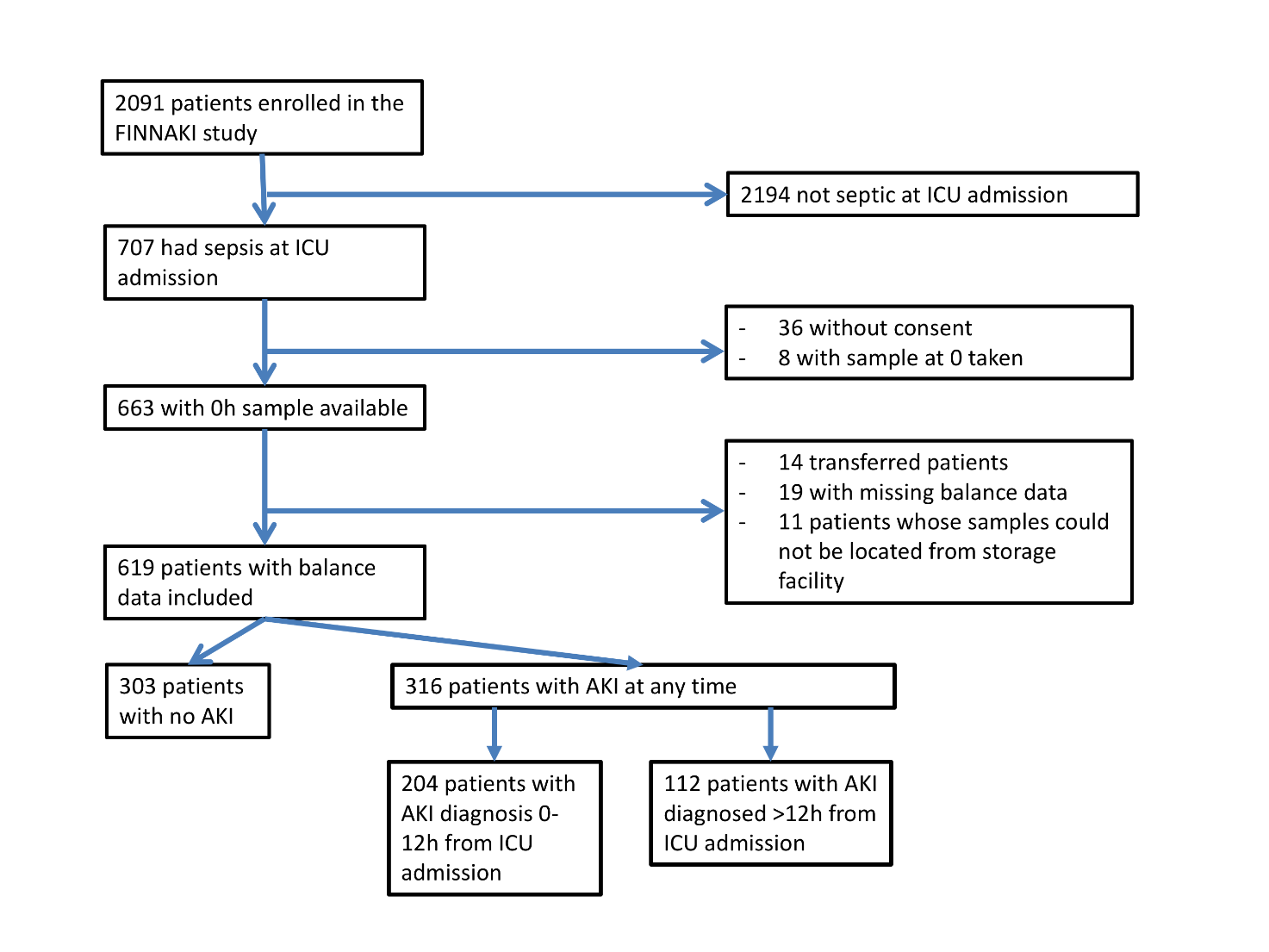


2.3 Additional file Table S1. Biomarkers and chronic diseases

| Biomarker level (ng/mL) at 0h (median [IQR]) |  | Syndecan-1 | Angiopoietin-2 | Soluble Thrombomodulin | Vascular adhesion protein-1 | Interleukin-6 |
| --- | --- | --- | --- | --- | --- | --- |
|  | Data available |  |  |  |  |  |
| Hypertension | 616 | 106.30 [58.90-207.70] | 6.64 [3.62-12.50] | 5.45 [3.95-7.21] | 241.00 [168.00-363.50] | 0.48 [0.13-4.09] |
| No hypertension |  | 112.40 [67.00-224.90] | 6.23 [3.34-11.62] | 5.25 [4.07-7.00] | 240.00 [172.00-331.00] | 0.75 [0.15-5.58] |
| *p* value |  | 0.216 | 0.692 | 0.859 | 0.532 | 0.154 |
| Coronary artery disease or ASO | 609 | 149.80 [71.35-254.80] | 7.82 [4.09-15.22] | 6.04 [4.30-7.84] | 263.00 [173.50-362.50] | 0.56 [0.17-3.03] |
| No coronary artery disease or ASO |  | 106.00 [60.50-191.28] | 6.06 [3.34-11.57] | 5.16 [3.99-6.98] | 239.00 [170.00-337.50] | 0.56 [0.13-5.08] |
| *p* value |  | 0.009 | 0.032 | 0.079 | 0.246 | 0.796 |
| Chronic heart failure | 613 | 112.20 [56.10-222.50] | 6.26 [3.64-15.03] | 4.99 [3.80-7.13] | 303.00 [203.00-418.00] | 0.36 [0.12-2.62] |
| No chronic heart failure |  | 108.50 [61.50-207.08] | 6.31 [3.37-11.75] | 5.37 [4.05-7.12] | 237.50 [169.50-332.50] | 0.66 [0.14-4.81] |
| *p* value |  | 0.873 | 0.529 | 0.252 | 0.003 | 0.198 |
| COPD | 612 | 100.00 [56.40-194.10] | 7.20 [4.01-14.61] | 5.46 [3.60-6.72] | 284.00 [195.00-393.00] | 0.25 [0.12-3.22] |
| No COPD |  | 109.70 [62.85-215.75] | 6.16 [3.33-11.89] | 5.32 [4.08-7.15] | 239.00 [170.00-337.00] | 0.67 [0.15-5.36] |
| *p* value |  | 0.427 | 0.220 | 0.201 | 0.060 | 0.009 |
| Chronic kidney disease (GFR<60mL/min/1.73m²) | 615 | 196.15 [73.23-285.30] | 7.29 [4.72-12.97] | 7.43 [5.46-9.88] | 295.50 [203.25-437.25] | 0.45 [0.13-3.50] |
| No chronic kidney disease |  | 107.60 [59.75-196.45] | 6.26 [3.34-12.01] | 5.15 [3.98-6.97] | 240.00 [169.00-337.00] | 0.57 [0.14-4.84] |
| *p* value |  | 0.021 | 0.194 | <0.001 | 0.010 | 0.449 |
| Diabetes | 619 | 110.30 [55.30-218.18] | 6.79 [4.04-13.71] | 5.88 [4.52-7.87] | 309.00 [209.00-424.25] | 0.37 [0.12-3.43] |
| No diabetes |  | 108.90 [63.90-214.35] | 6.23 [3.24-11.81] | 5.09 [3.90-6.81] | 225.00 [166.00-317.50] | 0.67 [0.15-4.96] |
| *p* value |  | 0.897 | 0.146 | <0.001 | <0.001 | 0.131 |
| Pre ICU chronic corticosteroid use | 615 | 127.10 [74.00-296.45] | 6.30 [3.59-10.27] | 5.79 [4.34-7.87] | 195.00 [147.00-295.50] | 0.74 [0.12-5.01] |
| No pre ICU chronic corticosteroid use |  | 108.50 [60.10-207.58] | 6.44 [3.37-12.67] | 5.17 [3.99-7.06] | 246.00 [172.75-356.50] | 0.56 [0.15-4.75] |
| *p* value |  | 0.048 | 0.511 | 0.038 | 0.001 | 0.808 |
| Pre ICU immunosuppression | 614 | 116.40 [71.90-437.38] | 6.21 [3.15-11.17] | 5.84 [4.15-9.43] | 211.50 [148.25-275.00] | 0.42 [0.12-3.46] |
| No pre ICU immunosuppression |  | 108.85 [58.90-207.08] | 6.39 [3.45-12.39] | 5.22 [4.03-7.10] | 245.00 [171.00-355.25] | 0.60 [0.15-4.94] |
| *p* value |  | 0.046 | 0.804 | 0.077 | 0.010 | 0.264 |

ASO; arteriosclerosis obliterans, COPD; *c*hronic obstructive pulmonal disease, ICU; intensive care unit

2.4 Additional file Figure S2.

Sequential samples subanalysis including 40 patients with biomarkers measured every 12 hrs until 36 hrs.





2.5 Additional file Table S2. Correlation between biomarkers

| Biomarker at 0h | Syndecan-1 | Angiopoietin-2 | soluble Thrombomodulin | Vascular adhesion protein-1 | Interleukin-6 |
| --- | --- | --- | --- | --- | --- |
| Syndecan-1 | - | 0.326/<0.001 | 0.406/<0.001 | 0.078/0.051 | 0.103/0.010 |
| Angiopoietin-2 | - | - | 0.345/<0.001 | 0.089/0.028 | 0.199/<0.001 |
| soluble Thrombomodulin | - | - | - | 0.119/0.003 | 0.093/0.021 |
| Vascular adhesion protein-1 | - | - | - | - | -0.289/<0.001 |

Spearman's rho Correlation Coefficient/p-value

2.6 Additional File Table S3. Multivariable linear regression models predicting log Fluid input on Day 0 normalized to hours (*n*=616)

| Model #, (Δ*R*^2^) | Variable | β, (95% CI) | *p* value |
| --- | --- | --- | --- |
| Model 1, (0.003) | Log Syndecan-1 | 0.059 (-0.014, 0.132) | 0.114 |
|  | Log Interleukin-6 | 0.392 (0.319, 0.465 | <0.001 |
|  |  |  |  |
| Model 2 (0.004) | Log Angiopoietin-2 | -0.068 (-0.143, 0.006) | 0.071 |
|  | Log Interleukin-6 | 0.413 (0.339, 0.487) | <0.001 |
|  |  |  |  |
| Model 3 (0.001) | Log soluble Thrombomodulin | 0.036 (-0.037, 0.109) | 0.331 |
|  | Log Interleukin-6 | 0.395 (0.321, 0.468) | <0.001 |
|  |  |  |  |
| Model 4 (0.018) | Log Vascular adhesion protein-1 | -0.139 (-0.213, -0.064) | <0.001 |
|  | Log Interleukin-6 | 0.362 (0.287, 0.436) | <0.001 |

Δ*R*^2^ stand for the increase in *R*^2^ compared to a model with only Interleukin-6 as the predictor (*R*^2^ = 0.142). β stands for the standardized regression coefficient.

2.7 Additional File Table S4. Multivariable linear regression models predicting Cumulative balance %/weight (*n*=616)

| Model #, (Δ*R*^2^) | Variable | β, (95% CI) | *p* value |
| --- | --- | --- | --- |
| Model 1, (0.007) | Log Syndecan-1 | 0.083 (0.006, 0.160) | 0.034 |
|  | Log Interleukin-6 | 0.334 (0.258, 0.411) | <0.001 |
|  |  |  |  |
| Model 2 (0.004) | Log Angiopoietin-2 | -0.063 (-0.140, 0.014) | 0.109 |
|  | Log Interleukin-6 | 0.357 (0.279, 0.435) | <0.001 |
|  |  |  |  |
| Model 3 (0.004) | Log soluble Thrombomodulin | 0.062 (-0.015, 0.138) | 0.114 |
|  | Log Interleukin-6 | 0.337 (0.260, 0.413) | <0.001 |
|  |  |  |  |
| Model 4 (0.016) | Log Vascular adhesion protein-1 | -0.130 (-0.209, -0.052) | 0.001 |
|  | Log Interleukin-6 | 0.312 (0.234, 0.390) | <0.001 |

Δ*R*^2^ stand for the increase in *R*^2^ compared to a model with only Interleukin-6 as the predictor (*R*^2^ = 0.121). β stands for the standardized regression coefficient.

2.8 Additional file Table S5. Biomarker levels and received colloids and acute kidney injury

| Biomarker level (ng/mL) at 0h (median [IQR]) | AKI at any time (n=316) | No AKI (n=303) | p value |
| --- | --- | --- | --- |
| Syndecan-1 | 136.20 [68.65-272.80] | 91.40 [55.30-157.30] | <0.001 |
| Angiopoietin-2 | 7.31 [4.08-15.19] | 5.53 [2.99-9.92] | <0.001 |
| soluble Thrombomodulin | 6.06 [4.48-8.32] | 4.61 [3.69-5.97] | <0.001 |
| Vascular adhesion protein-1 | 232.50 [166.00-335.00] | 255.00 [177.00-356.00] | 0.103 |
| Interleukin-6 | 1.03 [0.18-8.95] | 0.36 [0.12-3.06] | <0.001 |
| Received colloids (ml) at day 0 (median [IQR]) | 500 [0.00-1000.00] | 0.00 [0.00-500.00] | <0.001 |

IQR; interquartile range, AKI; acute kidney injury

References

1. Teng YH-F, Aquino RS, Park PW. Molecular functions of syndecan-1 in disease. Matrix Biol J Int Soc Matrix Biol. 2012 Jan;31(1):3–16.

2. Gonzalez Rodriguez E, Ostrowski SR, Cardenas JC, Baer LA, Tomasek JS, Henriksen HH, et al. Syndecan-1: A Quantitative Marker for the Endotheliopathy of Trauma. J Am Coll Surg. 2017 Sep;225(3):419–27.

3. Johansen ME, Johansson PI, Ostrowski SR, Bestle MH, Hein L, Jensen ALG, et al. Profound endothelial damage predicts impending organ failure and death in sepsis. Semin Thromb Hemost. 2015 Feb;41(1):16–25.

4. Puskarich MA, Cornelius DC, Tharp J, Nandi U, Jones AE. Plasma syndecan-1 levels identify a cohort of patients with severe sepsis at high risk for intubation after large-volume intravenous fluid resuscitation. J Crit Care. 2016;36:125–9.

5. Lukasz A, Hillgruber C, Oberleithner H, Kusche-Vihrog K, Pavenstädt H, Rovas A, et al. Endothelial glycocalyx breakdown is mediated by angiopoietin-2. Cardiovasc Res. 2017 May 1;113(6):671–80.

6. Parikh SM, Mammoto T, Schultz A, Yuan H-T, Christiani D, Karumanchi SA, et al. Excess circulating angiopoietin-2 may contribute to pulmonary vascular leak in sepsis in humans. PLoS Med. 2006 Mar;3(3):e46.

7. Fisher J, Douglas JJ, Linder A, Boyd JH, Walley KR, Russell JA. Elevated Plasma Angiopoietin-2 Levels Are Associated With Fluid Overload, Organ Dysfunction, and Mortality in Human Septic Shock. Crit Care Med. 2016 Nov;44(11):2018–27.

8. Mikacenic C, Hahn WO, Price BL, Harju-Baker S, Katz R, Kain KC, et al. Biomarkers of Endothelial Activation Are Associated with Poor Outcome in Critical Illness. PloS One. 2015;10(10):e0141251.

9. Katayama S, Nunomiya S, Koyama K, Wada M, Koinuma T, Goto Y, et al. Markers of acute kidney injury in patients with sepsis: the role of soluble thrombomodulin. Crit Care Lond Engl. 2017 Aug 25;21(1):229.

10. van der Heijden M, Pickkers P, van Nieuw Amerongen GP, van Hinsbergh VWM, Bouw MPWJM, van der Hoeven JG, et al. Circulating angiopoietin-2 levels in the course of septic shock: relation with fluid balance, pulmonary dysfunction and mortality. Intensive Care Med. 2009 Sep;35(9):1567–74.

11. Chao T-H, Tsai W-C, Chen J-Y, Liu P-Y, Chung H-C, Tseng S-Y, et al. Soluble thrombomodulin is a paracrine anti-apoptotic factor for vascular endothelial protection. Int J Cardiol. 2014 Mar 15;172(2):340–9.

12. Sapru A, Calfee CS, Liu KD, Kangelaris K, Hansen H, Pawlikowska L, et al. Plasma soluble thrombomodulin levels are associated with mortality in the acute respiratory distress syndrome. Intensive Care Med. 2015 Mar;41(3):470–8.

13. Pannecoeck R, Serruys D, Benmeridja L, Delanghe JR, van Geel N, Speeckaert R, et al. Vascular adhesion protein-1: Role in human pathology and application as a biomarker. Crit Rev Clin Lab Sci. 2015;52(6):284–300.

14. Salmi M, Jalkanen S. Ectoenzymes in leukocyte migration and their therapeutic potential. Semin Immunopathol. 2014 Mar;36(2):163–76.
